# Supplementary material for: CASi: A framework for cross-timepoint analysis of single-cell RNA sequencing data
Source: Sci Rep. 2024 May 9;14:10633. doi: 10.1038/s41598-024-58566-x (PMC11082156; doi:10.1038/s41598-024-58566-x)
Supplement: Supplementary file 1 — Supplementary Information. [file 41598_2024_58566_MOESM1_ESM.pdf]

Methods

Artificial neural networks (ANNs)

Denote the scRNA-seq expression matrix of the training data by $\boldsymbol{X}_0$ where $\boldsymbol{X}_0$ is a p by n_0 matrix with p being the total number of measured genes and n_0 is the number of cells, and the corresponding training cell label, $\boldsymbol{Y}_0$, is a n_0 by 1 vector. Similarly, denote the expression matrix of the testing data by $\boldsymbol{X}_1$, which has dimensions p by n_1 and the labels by $\boldsymbol{Y}_1$. After the standard min-max normalization, we select the top 2000 most variable genes from X_0 and the same set of features from $\boldsymbol{X}_1$. Next, using Keras (Chollet, 2016), an open-source software library that provides a Python interface for artificial neural networks, we train a neural network model with one input layer, one output layer, and three hidden layers:

$$
\begin{aligned}
\Pr\left(\boldsymbol{y_0} \mid \boldsymbol{X}, \boldsymbol{\theta}\right) &= \eta\left(\boldsymbol{Z}_{\text{out}}\boldsymbol{W}_{\text{out}} + \boldsymbol{\beta}_{\text{out}}\right) \\
\boldsymbol{Z}_{\text{out}} &= \sigma\left(\boldsymbol{Z}_3\boldsymbol{W}_3 + \boldsymbol{\beta}_3\right) \\
\boldsymbol{Z}_3 &= \sigma\left(\boldsymbol{Z}_2\boldsymbol{W}_2 + \boldsymbol{\beta}_2\right) \\
\boldsymbol{Z}_2 &= \sigma\left(\boldsymbol{Z}_1\boldsymbol{W}_1 + \boldsymbol{\beta}_1\right) \\
\boldsymbol{Z}_1 &= \sigma\left(\boldsymbol{X}_0\boldsymbol{W}_0 + \boldsymbol{\beta}_0\right)
\end{aligned}
$$

The parameter set $\theta = \{\boldsymbol{W}_0, \cdots, \boldsymbol{W}_3, \boldsymbol{\beta}_0, \cdots, \boldsymbol{\beta}_3, \boldsymbol{\beta}_{\text{out}}, \boldsymbol{W}_{\text{out}}\}$ will be estimated during the training process. And z_l for $l = \{1, 2, 3\}$ are the hidden neurons with corresponding weight $\boldsymbol{W}_1$, and bias $\boldsymbol{\beta}_1.\sigma(\cdot)$ is the activation function, which can be a sigmoid, a rectified linear unit (ReLU), a hyperbolic tangent, etc. We choose to use the ReLU function in our hidden layers because the neural networks based on an ReLU function are generally easier to train and can avoid the vanishing gradient problem during optimization (Eckle and Schmidt-Hieber, 2019); it is mathematically expressed as $\sigma_{\text{ReLU}}(x) = \max(x, 0)$. The SoftMax function will be used in the output layer. This is because the number of output categories is more than two, and it converts the values of the output layer into the predicted probabilities of each label. The number of neurons in the three hidden layers is selected as $\{256, 128, 64\}$. The model is trained using a stochastic gradient descent (SGD)-based algorithm with the mean squared error loss function $\mathcal{L}\left(\Pr(\boldsymbol{y_0}), Y_0\right) = \|\Pr(\boldsymbol{y_0}) - Y_0\|^2$. We use Adam as the optimization algorithm (Kingma and Ba, 2014), and the mini-batch training strategy (Li et al., 2014), which randomly trains a small proportion of samples and validates the rest of the samples in each iteration to improve training efficiency. By monitoring the loss, we implement the early stopping rule in Keras to avoid overfitting. Once the model performance stops improving for a couple epochs, the training process will stop. Additionally, to further prevent the overfitting issue, we add a dropout step with the dropping rate of 0.4 for each hidden layer to randomly drop units from the neural network during training. A one-time training process of ANN is shown in Fig.1.

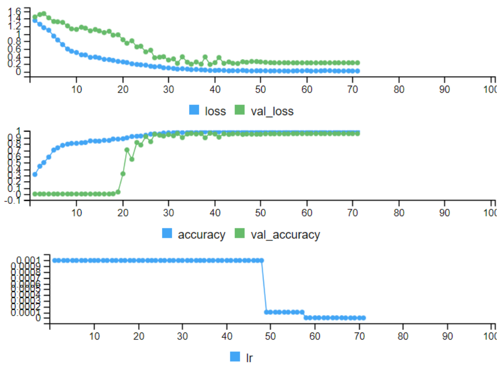

Fig. 1: ANN training process.

Simulation

Three scenarios

The simulation data are generated by assuming three sampling time points: t_0, t_1, t_2. To fully evaluate our method, we designed three scenarios: 1) t_0, t_1, and t_2 data contain the same cell types but with different cell type compositions; 2) a cell type in t_0 disappears in t_1 and t_2 data, i.e., t_0 data have one more cell type than t_1 and t_2 data; 3) a new cell type appears in t_1 and t_2 data, i.e., t_1 and t_2 data have one more cell type than t_0 data. We obtain a publicly available dataset of peripheral blood mononuclear cells (PBMC) (Zheng et al., 2017), containing more than 60,000 sorted cells from eight immune cell types. We randomly extract cells from five cell types and use different cell type compositions for different scenarios. Detailed cell compositions are presented in Table 1.

Table 1. Simulation: cell type compositions

	Scenario 1			Scenario 2			Scenario 3		
Cell type	t1	t2	t3	t1	t2	t3	t1	t2	t3
B cells	400	200	100	400	200	0	0	200	300
cd14	200	200	100	200	200	100	200	200	100
cd56 NK	400	300	200	400	300	200	400	300	200
memory T	500	400	300	500	400	300	500	400	300
cytotoxic T	500	400	300	500	400	300	500	400	300

Generate tDEGs

To evaluate the performance of identifying tDEGs, we conduct extensive simulations by manually creating tDEGs and calculating the true discovery rate. Considering that different types of cells will have very different gene expression profiles, we only extract monocyte cells from the PBMC data to do simulation. We draw 2000 genes and 900 monocyte cells. Among 2000 genes, 300 genes are randomly chosen to be tDEGs. First, we add group effect. Among 900 cells, half of the cells are assigned to be responders by multiplying the baseline gene expression by $Unif(1.5, 2)$, i.e., a uniform distribution of min $= 1.5$ and max $= 2$, while the other half of the cells are assigned to be non-responders and their baseline gene expressions stay the same. Next, we divided the whole data into three time points (t_0, t_1, and t_2); namely we will have 150 responder cells and 150 non-responder cells at each time point. We manually assign 300 genes to be the tDEGs

by adding the time effect. We design three levels of time effect: weak, medium, and strong. For the weak time effect scenario, the baseline gene expression of t_0, t_1, and t_2 cells are multiplied by $Unif(0.8, 1)$, $Unif(1, 1.2)$, and $Unif(1.2, 1.4)$, respectively. For the medium time effect scenario, the baseline gene expression of t_0, t_1, and t_2 cells are multiplied by $Unif(0.4, 0.6)$, $Unif(1, 1.2)$, and $Unif(1.6, 1.8)$, respectively. For the strong time effect scenario, the baseline gene expression of t_0, t_1, and t_2 cells are multiplied by $Unif(0.4, 0.6)$, $Unif(1.2, 1.4)$, and $Unif(2, 2.2)$, respectively.

Result figures

Simulation study

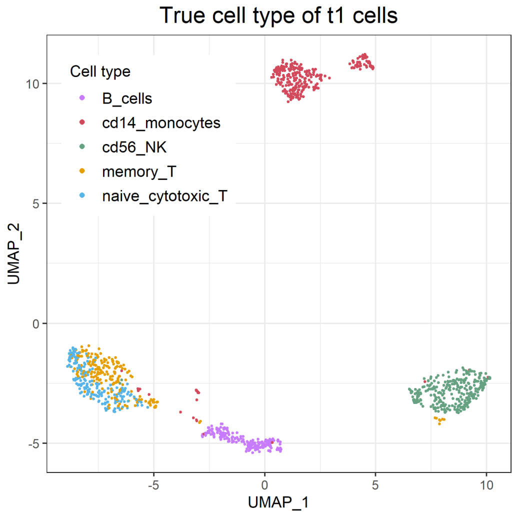

Fig. 2: The UMAP plot of t_1 data cell types.

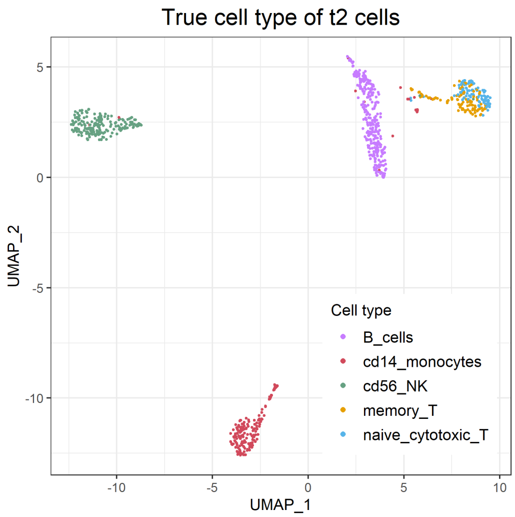

Fig. 3: The UMAP plot of t_2 data cell types.

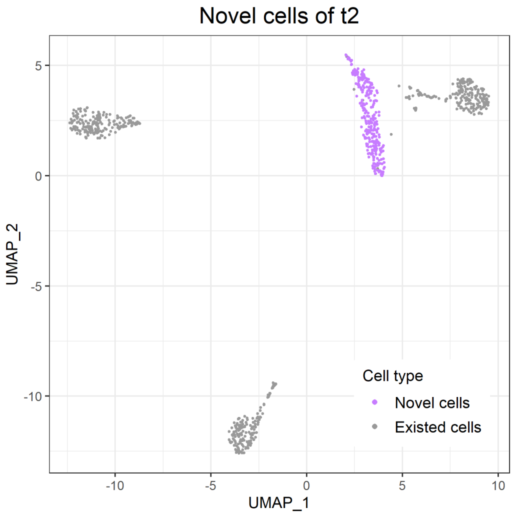

Fig. 4: The UMAP plot of t_2 data, with the purple cluster representing the novel cell type and the grey cluster representing existed cell types.

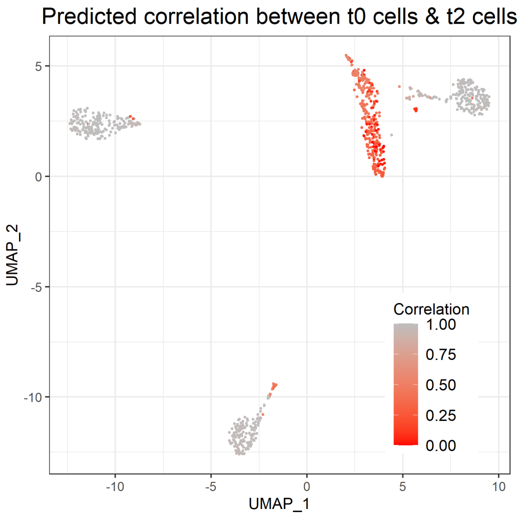

Fig. 5: The UMAP plot of t_2 data, showing the correlation levels between existing cell types in t_0 data and new cell types in t_2 data.

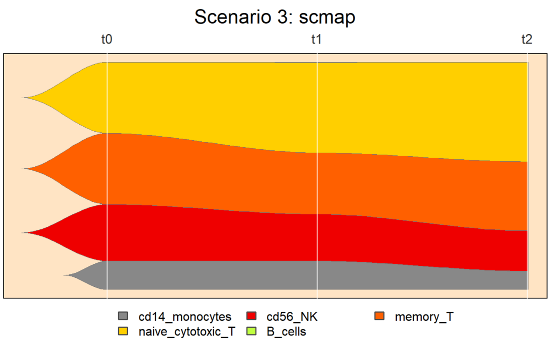

Fig. 6: The scmap fish plot of scenario 3 in which a novel cell type appears. We use B cells as the novel cell type which shows in green. And scmap fails to capture any novel cell.

A real-world multi-timepoint dataset

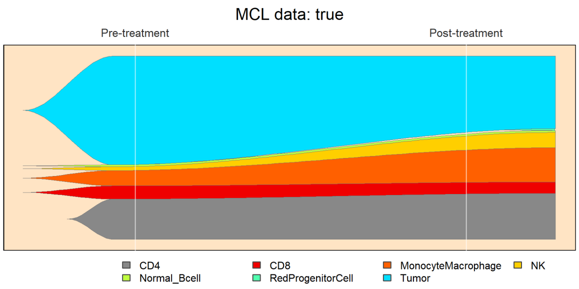

Fig. 7: MCL real-world data: the fish plots of the true cell population.

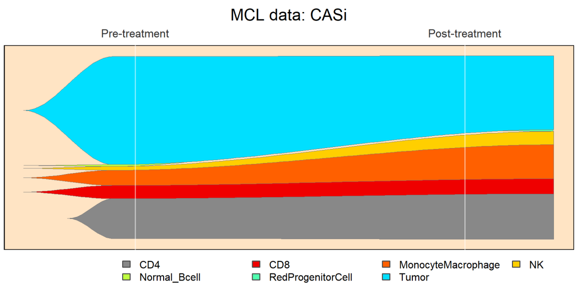

Fig. 8: MCL real-world data: the fish plots of the annotated cell population by CASi.

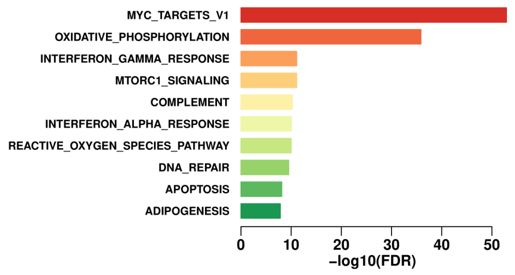

Fig. 9: MCL real-world data: the hallmark pathway results of enrichment analysis using 500 temporal differentially expressed genes identified by CASi.

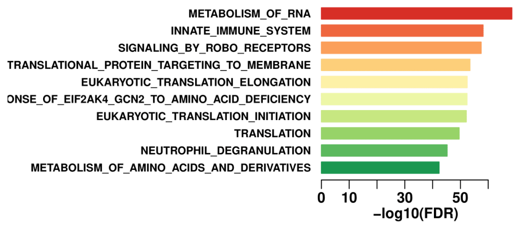

Fig. 10: MCL real-world data: the reactome pathway results of enrichment analysis using 500 temporal differentially expressed genes identified by CASi.

Discussion

CASi avoids the overclustering issue

The first step of CASi uses the neural network classifier to achieve cross-time points cell annotation with high accuracy. And as a supervised learning method, it efficiently avoids the overclustering issue. Using the same scenario settings of the simulation, we compare ARI (adjusted rand index) of unsupervised clustering implemented in the Seurat package with ARI of CASi using supervised clustering. The results of three scenarios are shown in Fig.11. It can be observed that our method's ARI increases with the cell number increasing, while the Seurat ARI decreases with the cell number increasing. This indicates that an overclustering issue does exist in unsupervised clustering methods, such as Seurat, and supervised clustering methods, such as CASi, are able to avoid this issue.

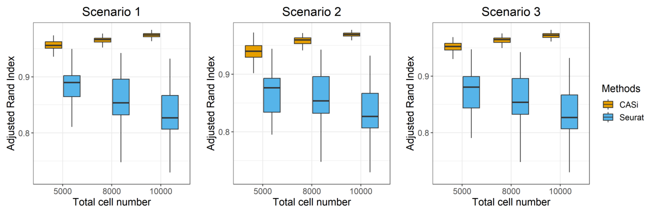

Fig. 11: Adjusted rand index (ARI) results of simulation study using different total cell numbers based on 200 repetitions.

References

F. Chollet. Building autoencoders in keras. *The Keras Blog*, 14, 2016.

K. Eckle and J. Schmidt-Hieber. A comparison of deep networks with relu activation function and linear spline-type methods. *Neural Networks*, 110:232–242, 2019.

D. P. Kingma and J. Ba. Adam: A method for stochastic optimization. *arXiv preprint arXiv:1412.6980*, 2014.

M. Li, T. Zhang, Y. Chen, and A. J. Smola. Efficient mini-batch training for stochastic optimization. In *Proceedings of the 20th ACM SIGKDD international conference on Knowledge discovery and data mining*, pages 661–670, 2014.

G. X. Zheng, J. M. Terry, P. Belgrader, P. Ryvkin, Z. W. Bent, R. Wilson, S. B. Ziraldo, T. D. Wheeler, G. P. McDermott, J. Zhu, et al. Massively parallel digital transcriptional profiling of single cells. *Nature communications*, 8(1):1–12, 2017.
